# Incremental Value of PI-RADS v2.1 for Adverse Pathology at Radical Prostatectomy: A Retrospective 1.5-T MRI Cohort

**DOI:** 10.3390/curroncol33090554

**Published:** 2026-09-11

**Authors:** Yunus Kayali, Onur Erdemoglu, Yigit Kudret Akyol, Melih Colak, Alpcan Torun, Fatih Bicaklioglu, Bilal Eryildirim

**Affiliations:** Department of Urology, University of Health Sciences, Istanbul Kartal Dr. Lutfi Kirdar City Hospital, 34865 Istanbul, Turkey; dr.yunuskayali@gmail.com (Y.K.); onurerdemogluonur@gmail.com (O.E.); yigitakyolmd@gmail.com (Y.K.A.); melihcolak64@gmail.com (M.C.); alpcantorun@gmail.com (A.T.); bilaleryildirim@yahoo.com (B.E.)

**Keywords:** prostate cancer, radical prostatectomy, adverse pathology, multiparametric magnetic resonance imaging, 1.5 Tesla, PI-RADS, systematic biopsy, PSA density, prediction

## Abstract

The incremental value of PI-RADS for predicting adverse pathology at radical prostatectomy in men diagnosed through systematic biopsy remains uncertain. We assessed whether PI-RADS improves discrimination beyond age, MRI-derived prostate-specificantigen density (PSAD), biopsy Grade Group, and quantitative biopsy tumour burden. This retrospective study included 284 men undergoing 1.5-T mpMRI, systematic biopsy, and radical prostatectomy between January 2020 and January 2026. Adverse pathology comprised prostatectomy Grade Group ≥ 3 and/or stage ≥ pT3a. Three logistic models were compared: age/PSAD/biopsy grade/tumour burden; age/PSAD/PI-RADS; and their combination. Internal validation used 2000 bootstrap samples. Adverse pathology occurred in 143 patients (50.4%). Apparent AUCs were 0.790 (95% CI 0.737–0.843), 0.713 (0.653–0.773), and 0.799 (0.747–0.851); optimism-corrected AUCs were 0.781, 0.703, and 0.787. Adding PI-RADS ≥ 4 yielded an adjusted OR of 1.996 (95% CI 1.150–3.465) and an apparent AUC difference of 0.009 (95% CI −0.015 to 0.033; descriptive DeLong *p* = 0.469). The combined model had a corrected calibration slope of 0.915 and Brier score of 0.187. PI-RADS from routine 1.5-T mpMRI was independently associated with adverse pathology, but its incremental discrimination beyond clinical and systematicbiopsy variables was small and uncertain. These findings concern a surgery-selected systematicbiopsy pathway, do not estimate the overall value of MRI, and require external validation.

## Simple Summary

Magnetic resonance imaging helps assess prostate cancer. In men diagnosed through systematic biopsy, it remains unclear whether MRI-based PI-RADS scores add to clinical and biopsy information in predicting unfavourable pathological findings at radical prostatectomy. We examined 284 men who underwent radical prostatectomy to assess whether the PI-RADS imaging score improved prediction of unfavourable pathological findings beyond age, prostate-specificantigen density, biopsy grade, and the amount of cancer in biopsy tissue. Adding PI-RADS increased the area under the prediction curve from 0.790 to 0.799. PI-RADS was associated with unfavourable pathological findings, but the observed improvement in prediction was small and its magnitude remained uncertain. These results apply to the studied systematicbiopsy and surgery pathway and require testing in independent cohorts.

## 1. Introduction

Prostate cancer is a major contributor to cancer morbidity in men. In the United States, 333,830 diagnoses and 36,320 deaths are estimated for 2026 [1]. For men considering radical prostatectomy, counselling and treatment planning depend partly on the likelihood of high-grade or non-organ-confined disease. Biopsy and clinical assessment provide preoperative estimates, whereas the prostatectomy specimen establishes pathological grade and extent [2,3]. The practical question is how much each available source of information improves that estimate.

Multiparametric magnetic resonance imaging (mpMRI), reported using the Prostate Imaging Reporting and Data System (PI-RADS), supports lesion detection and biopsy planning [4]. Prospective studies and a systematic review established its role in identifying clinically significant cancer and directing biopsy [5,6,7,8]. Predicting adverse pathology after cancer has already been diagnosed is a different task. MRI has high specificity but only moderate sensitivity for local extension, and performance varies across clinical settings [9,10].

PI-RADS, PSA density (PSAD), biopsy Grade Group, and biopsy tumour burden may contribute overlapping information. MRI-inclusive models can improve prediction of extraprostatic extension [11], and PSAD can refine risk assessment alongside PI-RADS [12,13]. However, an association between PI-RADS and aggressive disease does not by itself establish incremental discrimination for adverse pathology beyond biopsy grade and tumour burden.

This issue is particularly pertinent in routine 1.5-T practice. MRI performance is affected by acquisition and interpretation quality [14,15,16,17], and current diagnostic pathways increasingly combine targeted and systematic sampling [18]. Evidence derived from expert-centre 3-T imaging or MRI-targeted biopsy cannot automatically be extrapolated to cohorts diagnosed exclusively by systematic sampling.

We evaluated men who underwent routine 1.5-T mpMRI reported using PI-RADS v2.1, systematic transrectal ultrasound-guided biopsy without MRI targeting, and radical prostatectomy. Our main objective was to quantify the incremental discrimination provided by PI-RADS classification for adverse pathology at radical prostatectomy beyond age, MRI-derived PSAD, systematicbiopsy Grade Group, and quantitative biopsy tumour burden. This comparison addresses the contribution of PI-RADS within the studied surgical pathway.

## 2. Materials and Methods

### 2.1. Study Design, Patient Selection, and Data Collection

**Figure 1:**
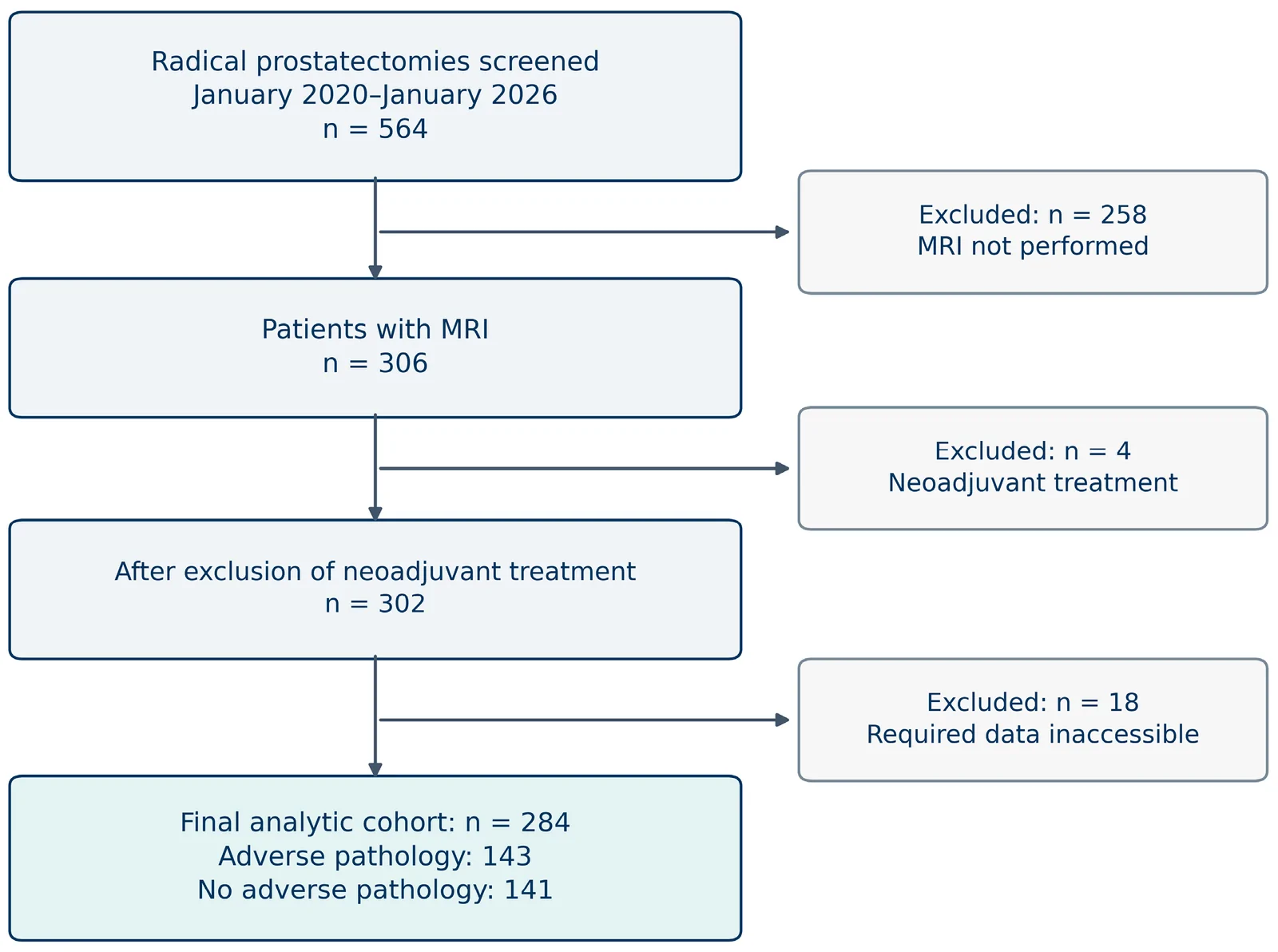
Derivation of the analytic cohort.

This retrospective, single-centre study screened all 564 men who underwent radical prostatectomy at the institution between January 2020 and January 2026. The study period reflects institutional use of PI-RADS v2.1 after its publication in 2019 [4]. Eligibility required histologically confirmed prostate adenocarcinoma, preoperative routine 1.5-T mpMRI reported using PI-RADS v2.1, systematicbiopsy findings, radicalprostatectomy pathology, and complete model predictors and outcomes. Of the 564 patients, 258 were excluded because MRI had not been performed. Of the remaining 306, four were excluded because they had received neoadjuvant treatment. Among the remaining 302, required study data could not be accessed for 18 patients. The final analytic cohort therefore comprised 284 patients. These sequential exclusions are shown in Figure 1. Whole-gland radicalprostatectomy pathology provided the reference standard for evaluating preoperative predictors.

Demographic, clinical, radiological, biopsy, surgical, and pathological data were obtained from electronic medical records and institutional databases and analysed in de-identified form. The study was conducted in accordance with the Declaration of Helsinki and was approved by the Institutional Clinical Research Ethics Committee (decision no: 2026/010.99/30/4; 22 July 2026). The committee waived the requirement for informed consent because this retrospective, non-interventional study analysed existing anonymised clinical data without additional procedures or direct patient contact.

Reporting was prepared with reference to STROBE recommendations and TRIPOD + AI guidance for regression-based prediction models [19].

### 2.2. Clinical and mpMRI Variables

Clinical variables included age at surgery, serum prostate-specific antigen (PSA) at diagnosis, clinical T stage, and prostate volume measured on preoperative mpMRI. Clinical T stage was extracted from the rectal-examination field. cT1c denotes non-palpable cancer diagnosed by needle biopsy; cT2a and cT2b denote palpable, organ-confined involvement of at most half or more than half of one side, respectively. cT2c denotes bilateral involvement. The source categories were cT1c, cT2a, cT2b, and ≥cT2c; the last category does not distinguish cT2c from more advanced clinical stages. PSA density was calculated as serum PSA divided by mpMRI-derived prostate volume.

All patients underwent preoperative mpMRI on a 1.5-T Magnetom Avanto scanner (Siemens Healthcare, Erlangen, Germany). No endorectal coil was used. The same protocol was used for all patients: axial, sagittal, and coronal T2-weighted imaging; axial diffusion-weighted imaging with apparent diffusion coefficient (ADC) maps; axial fat-suppressed T2-weighted imaging; coronal T1-weighted imaging; and axial dynamic contrast-enhanced MRI. For dynamic contrast-enhanced imaging, a gadolinium-based contrast agent was administered as a bolus at 0.1 mmol/kg body weight, followed by a 20 mL saline flush.

Axial diffusion-weighted imaging was performed using an echo-planar imaging sequence with b values of 50, 500, 800, 1000, and 1200 s/mm^2^, and ADC maps were generated. The available protocol records did not distinguish which b-value images were directly acquired and which were calculated. Axial dynamic contrast-enhanced MRI was performed using a two-dimensional fast low-angle shot (2D FLASH) sequence (TR/TE, 4.9/2.4 ms) with 16 measurements after administration of the gadolinium-based contrast agent and saline flush. The temporal resolution per dynamic measurement could not be recovered from the available records.

In all patients, mpMRI was performed before prostate biopsy and radical prostatectomy. The examinations were interpreted by three radiologists, each with at least 3 years of experience in prostate MRI interpretation. At the time of mpMRI interpretation, radiologists had access to the serum PSA level and relevant clinical examination findings, when available. Because mpMRI preceded prostate biopsy and radical prostatectomy, histopathological information from biopsy and prostatectomy specimens was not available to the radiologists. For patients with multiple lesions, the highest PI-RADS score among all reported lesions was used as the patient-level PI-RADS score. MRI findings were interpreted according to PI-RADS v2.1 [4] and extracted from the original clinical reports. Recorded variables included PI-RADS category, number of reported lesions, largest lesion diameter, and radiological suspicion of extraprostatic extension. For descriptive evaluation, PI-RADS scores were categorised both along the ordinal scale (1–5) and as a binary grouping (≤3 vs. ≥4) as part of the study design. The main models retained PI-RADS ≥ 4 versus ≤3, and the ordinal sensitivity analysis used a numeric score from 1 to 5, with an odds ratio per one-category increase. No central blinded rereading or formal retrospective image-quality reassessment was performed. Because PSAD uses MRI-derived prostate volume, the reference model already incorporates MRI-derived information even without PI-RADS.

### 2.3. Systematic Biopsy Procedure and Pathology

Systematic prostate biopsies were performed under transrectal ultrasound guidance using the institutional diagnostic protocol. The standard diagnostic protocol consisted of a 12-core systematic biopsy, although the total number of cores could vary according to clinical circumstances. In selected patients, additional cores were obtained from sonographically suspicious areas, and extended or 24-core saturation sampling was performed when clinically indicated. Neither software-based MRI–ultrasound fusion-targeted biopsy nor cognitive MRI-targeted biopsy was performed during the study period; therefore, the preoperative biopsy dataset was derived exclusively from systematic TRUS-guided sampling.

Recorded biopsy variables included the total number of cores, number and percentage of positive cores, longest cancer length, total sampled core length, total cancer length, biopsy ISUP Grade Group, bilateral tumour involvement, perineural invasion, and high-grade prostatic intraepithelial neoplasia. The tumour-to-core-length ratio was derived by dividing the total cancer length by the total sampled core length and expressing the result as a percentage. Similarly, the positive-core percentage was defined as the ratio of positive cores to the total number of cores retrieved, also expressed as a percentage. All derived parameters were computed directly from the documented core measurements.

Biopsy Grade Group was dichotomised as 3–5 versus 1–2 for model construction.

### 2.4. RadicalProstatectomy Pathology and Outcome Definition

Radical prostatectomy specimens were evaluated in routine institutional pathology practice. ISUP Grade Group and pathological TNM stage were obtained from the original final pathology reports without central re-review. Adverse pathology was defined as radical prostatectomy ISUP Grade Group ≥ 3 (Gleason score ≥ 4 + 3) and/or pathological stage ≥ pT3a. The two components—Grade Group ≥ 3 and stage ≥ pT3a—were also analysed separately as secondary outcomes. The composite captures high-grade and non-organ-confined disease.

Pathological nodal status was not included in the primary endpoint because pelvic lymph-node dissection was not performed uniformly across the cohort. Nodal findings were therefore reported descriptively.

### 2.5. Prediction Models

Three multivariable binary logistic regression models were developed in this retrospective dataset. Model 1, the reference PSAD-plus-systematicbiopsy model, included age, MRI-derived PSAD, biopsy ISUP Grade Group (3–5 vs. 1–2), and the tumour-to-core-length ratio. Model 2, the age/PSAD/PI-RADS model, included age, MRI-derived PSAD, and PI-RADS ≥ 4 versus ≤3. Model 3 added PI-RADS ≥ 4 versus ≤3 to all predictors in Model 1. Because PSAD incorporated mpMRI-derived prostate volume, Model 1 was not entirely independent of MRI data, despite excluding lesion-level PI-RADS features. Consequently, the prespecified primary comparison was conducted between Model 3 and Model 1 to quantify the incremental predictive value of lesion-level PI-RADS classification beyond established clinical variables and systematic biopsy results, rather than to evaluate the global diagnostic utility of MRI.

### 2.6. Statistical Analysis

Statistical analyses used IBM SPSS Statistics for Windows, version 22.0 (IBM Corp., Armonk, NY, USA), and Python, version 3.12.13. The reported estimates were computed in Python using NumPy, pandas, SciPy, statsmodels, and custom routines for DeLong comparisons and bootstrap internal validation, and were verified using two separate numerical implementations. All *p* values were two-sided; 0.05 was the nominal significance threshold. Secondary analyses were exploratory, without multiplicity adjustment. Continuous variables are summarised as medians and interquartile ranges and were compared using the two-sided Mann–Whitney U test with tie and continuity correction. Categorical variables are summarised as counts and percentages. Pearson’s chi-square test without continuity correction was used when all expected cell counts were at least five. When any expected cell count was below five, Fisher’s exact test was used for 2 × 2 tables, and a conditional Monte Carlo test based on Pearson’s chi-square statistic, with 100,000 samples and fixed margins, was used for larger tables.

The primary outcome was analysed as a binary dependent variable. Univariable logistic regressions described unadjusted associations. Clinical T stage was treated categorically with cT1c as the reference. Odds ratios and their 95% confidence intervals were obtained by exponentiating the regression coefficient and its coefficient ±1.96 standard-error limits; two-sided Wald tests evaluated coefficients. The overall clinical-stage association used a three-degree-of-freedom Wald test. PSAD odds ratios represent a 0.10 ng/mL/cm^3^ increase; tumour-to-core-length ratio odds ratios represent a one-percentage-point increase. Age, PSAD, and tumour-to-core-length ratio remained continuous. Restricted cubic splines with knots at the 10th, 50th, and 90th percentiles assessed departures from linear log odds; likelihood-ratio tests assessed nonlinear terms jointly and separately. Knot locations were fixed within each cohort during resampling.

The original predictor choices reflected clinical relevance, univariable associations, redundancy among candidate variables, and observed model performance in this retrospective dataset. PSAD was used instead of separate PSA and prostatevolume terms, and the tumour-to-core-length ratio was used to represent quantitative biopsy burden. No automated forward, backward, or stepwise selection was applied. The three models described in Section 2.5 were fitted using unpenalised maximum likelihood estimation, with all specified predictors entered simultaneously. Bootstrap validation evaluated these fixed model structures; it could not fully account for optimism introduced by the earlier, partly data-informed selection of predictors. Clinical T stage was evaluated separately in a sensitivity analysis by adding cT ≥ 2 versus cT1c to Models 1 and 3.

Discrimination was quantified using the AUC, with 95% confidence intervals calculated using DeLong’s covariance estimator. Paired AUC differences and their 95% confidence intervals were also reported. Because model development and evaluation used the same cohort and Models 1 and 3 were nested, DeLong comparisons were treated as descriptive assessments of apparent discrimination rather than definitive tests of added predictive information. Likelihood-ratio tests compared nested models to assess the addition of PI-RADS or clinical T stage. An exploratory five-level categorical PI-RADS model was fitted using PI-RADS 1 as the reference category and four indicator terms for PI-RADS 2–5. Because the PI-RADS 1 category contained few patients, bootstrap internal validation was not performed for this exploratory model. Internal validation used 2000 nonparametric bootstrap samples generated by sampling analysis records with replacement. Within each cohort, the same set of bootstrap samples was used across model specifications. Each model undergoing internal validation was refitted in each bootstrap sample and evaluated in both that sample and the original cohort. Predictor sets were held fixed, and predictor selection was not repeated during resampling. Prediction error was quantified using the Brier score, defined as the mean squared difference between predicted probabilities and observed binary outcomes. For each bootstrap-fitted model, optimism in AUC was calculated as the AUC in the bootstrap sample minus the AUC in the original cohort. Mean optimism was subtracted from the apparent AUC. For the Brier score, the mean difference between the error in the original cohort and that in the bootstrap sample was added to the apparent score.

Calibration-in-the-large was estimated as the intercept of a logistic model with the prediction logit as an offset, and calibration slope was estimated from a logistic model using that logit as a predictor. Calibration plots displayed observed outcome proportions against mean predicted probabilities in ten risk groups, with Wilson 95% confidence intervals. These plots describe apparent calibration; corrected calibration summaries were obtained from bootstrap validation. Bootstrap replicates that did not yield stable, finite model or calibration estimates were excluded from the corresponding performance summaries. The number of usable replicates was reported for each model.

Threshold-based classification accuracy was not reported because no clinically justified decision threshold had been defined. Analyses used complete values for the included predictors and outcomes; no imputation was performed.

Sensitivity analyses assessed the robustness of the findings to alternative PI-RADS specifications, separate outcome components, additional adjustment for clinical T stage, and restriction by biopsy core count. In addition to the binary specification (PI-RADS ≥ 4 versus ≤3), PI-RADS was entered as a single numerical term coded from 1 to 5, assuming a linear association with the log odds of the outcome; the corresponding odds ratio represents a one-category increase. An exploratory version of the combined model treated PI-RADS as a five-level categorical variable, using four indicator variables with PI-RADS 1 as the reference category. This exploratory model was not internally validated because of the small number of patients in the PI-RADS 1 category. Separate analyses evaluated prostatectomy Grade Group ≥ 3 and pathological stage ≥ pT3a as individual outcomes. Analyses were also repeated among patients who underwent exactly 12-core systematic biopsy, with all models refitted within this subgroup.

### 2.7. Generative-AI Assistance

During the preparation of this manuscript, ChatGPT-5.6 Luna model (OpenAI, San Francisco, CA, USA; accessed on 3 August 2026) was utilised solely for language editing and text refinement. The AI tool was not involved in study design, statistical programming, data analysis, output validation, literature synthesis, or substantive interpretation. All statistical code execution, numerical calculations, and interpretations were performed independently by the authors, who take full responsibility for the final content, accuracy, and wording of the article.

## 3. Results

### 3.1. Cohort and Primary Outcome

The final analytic cohort comprised 284 patients. Adverse pathology was identified in 143 patients (50.4%), whereas 141 patients (49.6%) had no adverse pathological features.

Among the individual components of the primary composite endpoint, 20 patients (7.0%) had radical prostatectomy ISUP Grade Group ≥ 3 without pathological stage ≥ pT3a, 60 (21.1%) had pathological stage ≥ pT3a without ISUP Grade Group ≥ 3, and 63 (22.2%) had both adverse pathological features. Pelvic lymph-node dissection was performed in 120 patients (42.3%), of whom 111 had pN0 and nine had pN1 disease. All patients with pN1 disease also had pathological stage ≥ pT3a; therefore, inclusion of pN1 disease in the primary composite endpoint would not have reclassified any additional patient. Separate outcome-specific analyses are reported in Supplementary Table S1. For prostatectomy Grade Group ≥ 3, the M3 − M1 apparent ΔAUC was 0.013 (95% CI, −0.001 to 0.027; descriptive paired DeLong *p* = 0.065), and the adjusted OR for PI-RADS ≥ 4 versus ≤3 was 1.462 (95% CI, 0.743–2.877; Wald *p* = 0.271). For pathological stage ≥ pT3a, the corresponding ΔAUC was 0.003 (95% CI, −0.012 to 0.019; descriptive paired DeLong *p* = 0.662), and the adjusted PI-RADS OR was 1.659 (95% CI, 0.958–2.872; Wald *p* = 0.071).

### 3.2. Baseline Characteristics According to Adverse Pathology

Table 1 presents demographic, clinical, MRI, and systematicbiopsy characteristics according to adverse pathology. PI-RADS is reported both across its five individual categories and using the original ≤3/≥4 grouping.

Systematicbiopsy tumour burden is described using positive-core count and percentage, longest and total cancer lengths, and the tumour-to-core-length ratio. Biopsy Grade Group and the remaining categorical characteristics are reported with their full distributions in Table 1.

### 3.3. Univariable Associations

Univariable associations are reported as odds ratios with 95% confidence intervals in Table 2. For PSAD, the OR per 0.10 ng/mL/cm^3^ increase was 1.482 (95% CI, 1.226–1.792; *p* < 0.001). These analyses describe unadjusted associations and were not used to select predictors in the revision analyses.

### 3.4. Multivariable Models and Incremental Discrimination

Adjusted associations for all predictors in the three models are reported in Table 3, and the complete logisticregression coefficients and intercepts are provided in Supplementary Table S2. Model 1 contains age, MRI-derived PSAD and systematicbiopsy variables; Model 2 contains age, MRI-derived PSAD, and PI-RADS; and Model 3 adds PI-RADS to Model 1. PI-RADS ≥ 4 was associated with adverse pathology after adjustment for age, MRI-derived PSAD, biopsy Grade Group, and tumour-to-core-length ratio (adjusted OR, 1.996; 95% CI, 1.150–3.465; *p* = 0.014). The likelihood-ratio test for adding PI-RADS to Model 1 yielded *p* = 0.013.

**Figure 2:**
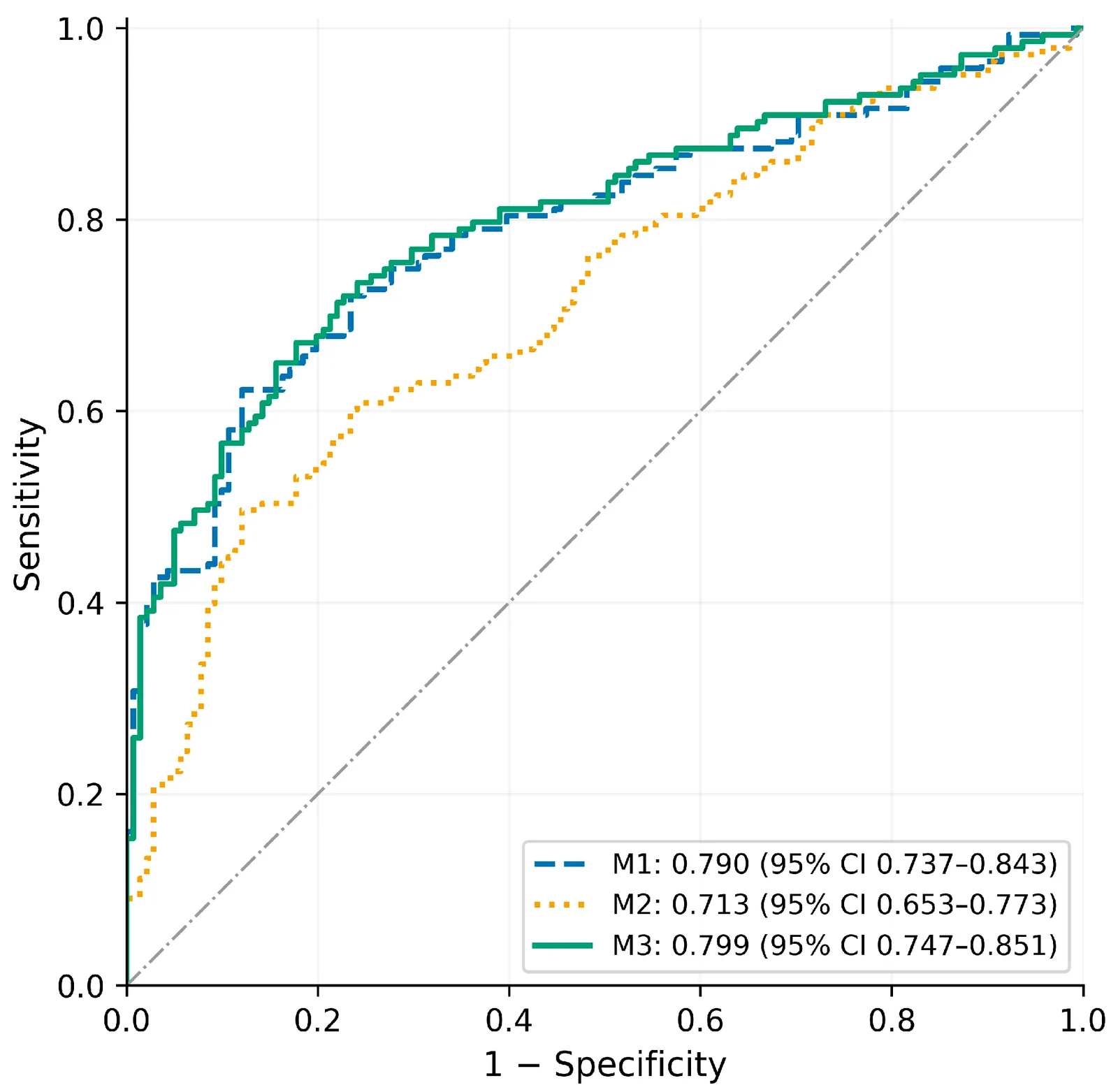
Apparent discrimination. Receiver operating characteristic (ROC) curves for predicting adverse pathology. Model curves are distinguished by colour and line style; corresponding apparent AUCs with 95% DeLong CIs are provided in the legend. The diagonal dashed line indicates chance performance. Model definitions: M1 (age, MRI-derived PSAD, biopsy Grade Group 3–5, and tumour-to-core-length ratio); M2 (age, MRI-derived PSAD, and PI-RADS ≥ 4); M3 (M1 plus PI-RADS ≥ 4). All models incorporate MRI-derived prostate volume via PSAD.

**Figure 3:**
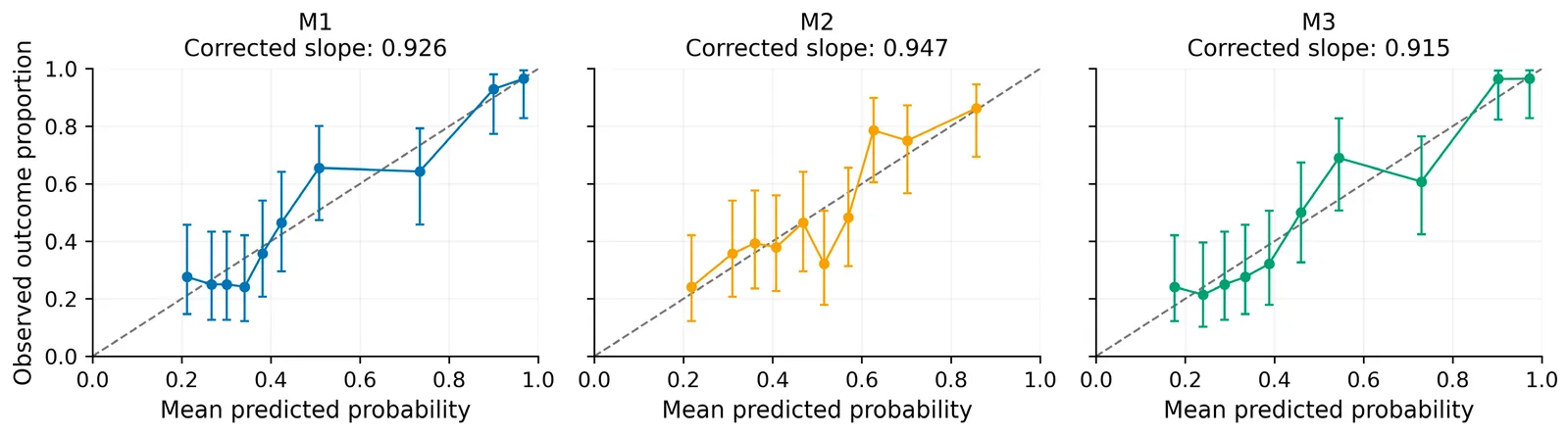
Apparent calibration. Calibration plots displaying observed outcome proportions against mean predicted probabilities across deciles of predicted risk. Error bars represent Wilson 95% confidence intervals, and the diagonal dashed line denotes ideal calibration. Plots depict apparent calibration performance; bootstrap-corrected calibration metrics are reported in Table 4. Model definitions: M1 (age, MRI-derived PSAD, biopsy Grade Group 3–5, and tumour-to-core-length ratio); M2 (age, MRI-derived PSAD, and PI-RADS ≥ 4); M3 (M1 plus PI-RADS ≥ 4). All models incorporate MRI-derived prostate volume via PSAD.

The apparent AUCs were 0.790 (95% CI, 0.737–0.843) for Model 1, 0.713 (0.653–0.773) for Model 2, and 0.799 (0.747–0.851) for Model 3. Corresponding optimism-corrected AUCs were 0.781, 0.703, and 0.787. The apparent AUC increase from Model 1 to Model 3 was 0.009 (95% CI, −0.015 to 0.033; descriptive paired DeLong *p* = 0.469). This interval did not establish equivalence or exclude a potentially meaningful incremental effect. Calibration and Brier scores are reported in Table 4, and paired AUC differences are reported in Table 5. In the full cohort, each one-category increase in PI-RADS was associated with an adjusted OR of 1.395 (95% CI, 1.066–1.827; *p* = 0.015); the ordinal combined model had an apparent AUC of 0.799 and an optimism-corrected AUC of 0.786 (Supplementary Table S3). In the exactly 12-core subgroup, the corresponding adjusted OR was 1.454 (95% CI, 1.088–1.942; *p* = 0.011), with an apparent AUC of 0.808 and an optimism-corrected AUC of 0.795 (Supplementary Table S3). Separate outcome-specific analyses are reported in Supplementary Table S1. The joint likelihood-ratio test for nonlinear terms was *χ*^2^(3) = 2.578 (*p* = 0.461), and adding cT ≥ 2 versus cT1c to Model 3 yielded *χ*^2^(1) = 0.461 (*p* = 0.497); these analyses did not provide evidence of statistically detectable nonlinear effects or an additional clinical T-stage contribution. The corresponding results, including the cT-adjusted PI-RADS increment, are reported in Supplementary Table S4. In the exactly 12-core subgroup, the binary models yielded apparent AUCs of 0.796, 0.712, and 0.807 for Models 1, 2, and 3, respectively. The M3 − M1 apparent AUC difference was 0.011 (95% CI, −0.015 to 0.038; descriptive paired DeLong *p* = 0.413), with optimism-corrected AUCs of 0.787, 0.702, and 0.795 (Supplementary Table S5). ROC curves are shown in Figure 2 and apparent calibration plots in Figure 3.

## 4. Discussion

In this cohort, incorporating PI-RADS category ≥ 4 into a baseline model comprising age, MRI-derived PSAD, biopsy grade, and tumour burden increased the apparent AUC from 0.790 to 0.799 (absolute difference: 0.009; 95% CI: −0.015 to 0.033). Nevertheless, PI-RADS maintained an independent association with adverse pathology in the multivariable model (adjusted OR: 1.996; 95% CI: 1.150–3.465). These findings clearly delineate two distinct clinical questions: whether PI-RADS remains independently associated with the outcome after multivariable adjustment, and to what extent it enhances patient risk discrimination. While its independent association was supported, any incremental contribution to discrimination was modest at best.

PSAD was associated with adverse pathology in the combined model, with an adjusted OR per 0.10 ng/mL/cm^3^ increase of 1.274 (95% CI, 1.046–1.551). Earlier work has examined the complementary use of PSAD and PI-RADS in prostate-cancer assessment [12,13]. In this study, prostate volume was obtained from MRI. Consequently, Model 1 already incorporated MRI-derived volumetric information through PSAD. The main comparison therefore assesses the incremental contribution of PI-RADS classification beyond a reference model that already includes MRI-derived prostate volume through PSAD.

Biopsy grade and quantitative tumour burden were associated with adverse pathology after adjustment for the other predictors. In Model 3, the adjusted OR was 11.830 (95% CI, 4.409–31.742) for biopsy Grade Group 3–5 versus 1–2 and 1.024 (95% CI, 1.006–1.042) per percentage-point increase in the tumour-to-core-length ratio. The magnitudes of these odds ratios cannot be directly compared because their predictor scales differ. Grade and sampled tumour extent describe different aspects of the biopsy findings. Their associations are compatible with the continued relevance of systematicbiopsy information discussed in the literature [18]. This cohort contains no MRI-targeted biopsies and cannot establish the comparative performance of systematic and targeted sampling.

The reference model incorporating age, MRI-derived PSAD, biopsy grade, and quantitative tumour burden showed greater apparent discrimination than the age/PSAD/PI-RADS model (AUC, 0.790 versus 0.713). This finding highlights the prognostic information available from systematicbiopsy grade and measured cancer extent once a tissue diagnosis has been established. The comparison should nevertheless be interpreted in relation to the prediction task. The diagnostic role of mpMRI in biopsy triage and lesion targeting is supported by studies conducted earlier in the diagnostic pathway [5,6,7,8]. Local staging also has limitations, particularly for small-volume extraprostatic disease [9,10]. Here, biopsy grade and measured cancer extent were already available before surgery. The comparison therefore evaluates the incremental contribution of PI-RADS to predicting adverse pathology at radical prostatectomy beyond clinical and biopsy information in men diagnosed through systematic biopsy. It does not assess the overall benefit of obtaining MRI.

Overlap among the information captured by PI-RADS, PSAD, biopsy grade, and biopsy tumour burden is one possible explanation for the observed AUC increment; this mechanism was not directly tested. A predictor may change estimated probabilities without substantially changing patient ordering, so an adjusted association and an AUC difference address distinct questions. The incremental contribution also depends on the reference predictors and outcome. In a diagnostic setting, Thompson et al. reported that adding PI-RADS to a model incorporating PSA, digital rectal examination, prostate volume, and age increased the AUC for detecting significant prostate cancer from 0.776 to 0.879 (*p* < 0.001) [20]. Their model addressed cancer detection before diagnostic biopsy, whereas ours predicted adverse pathology at radical prostatectomy using information that already included biopsy grade and quantitative tumour burden. Differences in endpoints, populations, sampling pathways, and reference-model information therefore limit direct comparison of the magnitude of the AUC increments.

The confidence interval for the AUC increment does not establish equivalence or exclude a clinically meaningful benefit. Both models were fitted and assessed in the same cohort; their paired DeLong comparison describes apparent performance and warrants caution because the models are nested. Bootstrap correction lowered the AUC estimates, and independent evaluation remains necessary. MRI-inclusive nomograms have been developed to predict side-specific extraprostatic extension [11]. In an external validation of side-specific prediction models, Diamand et al. found lower performance than previously reported, although selected models demonstrated useful discrimination and adequate calibration at lower predicted risks [21]. Alves et al. independently evaluated the Giganti–Coppola nomogram, which combines clinical and MRI parameters, and found that it overestimated the probability of extracapsular extension despite good discrimination [22]. These findings illustrate why discrimination and calibration both require assessment in independent populations. In a separate local-staging study, Soeterik et al. reported that mpMRI detected non-organ-confined disease with greater sensitivity than digital rectal examination (51% vs. 12%), but with lower specificity (82% vs. 97%) [23]. These studies support the contribution of MRI to local staging while demonstrating limitations in risk estimation and the trade-off between sensitivity and specificity. However, they evaluated anatomical extension or integrated prediction tools rather than the incremental contribution of PI-RADS to a model already containing detailed biopsy information. Differences in endpoints, predictor composition, and populations therefore limit direct comparison with our composite outcome of adverse grade or stage.

Clinically, these findings support considering reported PI-RADS alongside age, MRI-derived PSAD, biopsy grade, and quantitative tumour burden when counselling men with prostate cancer diagnosed through systematic biopsy who are proceeding to radical prostatectomy. Because clinical net benefit was not evaluated, the results do not establish a risk threshold for changing management. After adding clinical T stage (cT ≥ 2 versus cT1c) to Models 1 and 3, the apparent AUC difference between the models was 0.009 (95% CI, −0.014 to 0.033; descriptive DeLong *p* = 0.441; Supplementary Table S3). This represents a small observed increment, although the confidence interval leaves its magnitude uncertain. The recorded ≥ cT2c category did not distinguish cT2c from more advanced stages, limiting their separate evaluation. This sensitivity analysis assessed additional adjustment for clinical stage rather than validating an established risk tool. Future studies should evaluate clinical net benefit and the additional contribution of lesion characteristics, MRI suspicion of extraprostatic extension, and lesion–biopsy concordance in pathways incorporating MRI-targeted biopsy.

Our findings reflect routine clinical practice using 1.5-T mpMRI with a common acquisition protocol. Quality frameworks emphasise image-quality assessment, reader training and ongoing performance monitoring [14,15]. Poorer image quality has been associated with lower detection of clinically significant cancer [16], while protocol optimisation has improved image quality across centres [17]. In a 3-T setting, Faiena et al. associated PI-RADS 5 with adverse pathology and poorer biochemical recurrence-free survival in men with biopsy Gleason 3 + 4 disease [24]. Vargas et al. demonstrated limited detection of small high-grade foci on whole-mount comparison [25]. We performed no direct comparison of field strengths, central rereading or formal image-quality assessment; incomplete technical records also limited protocol characterisation. The modest AUC increment observed here therefore cannot be attributed specifically to field strength or directly generalised to 3-T examinations performed under different clinical and technical conditions. It should likewise not be interpreted as contradicting greater predictive performance reported elsewhere, where populations, outcomes and reference-model predictors may differ.

The strengths of this study include the use of radicalprostatectomy pathology as the reference standard, detailed systematicbiopsy grade and tumour-burden measurements, and bootstrap assessment of discrimination and calibration. Supplementary analyses were broadly consistent with the primary findings. Restricted cubic-spline analyses showed no statistically detectable departures from linear log odds for age, PSAD, or tumour-to-core-length ratio, while additional adjustment for cT ≥ 2 versus cT1c did not provide evidence of a statistically detectable change in the PI-RADS increment (Supplementary Table S3). Ordinal PI-RADS remained independently associated with adverse pathology in both the full cohort and the exactly 12-core subgroup, whereas restriction to exactly 12 systematic biopsy cores produced a similar, non-significant M3 − M1 AUC difference (Supplementary Tables S4 and S5). Separate grade and stage analyses were exploratory and did not establish statistically different PI-RADS associations across outcome components (Supplementary Table S1). Limitations include the retrospective, single-centre design restricted to men undergoing radical prostatectomy, which introduces referral, spectrum, and verification biases and excludes patients managed through other pathways. MRI examinations were interpreted during routine care without central rereading or formal retrospective quality assessment. PI-RADS was dichotomised in the primary analysis; ordinal analyses assessed robustness under a constant per-category log-odds assumption, whereas the separate five-category exploratory model was limited by the small number of PI-RADS 1 cases and was not internally validated. Because predictor selection was partly data-informed, bootstrap validation of the specified model structures may not fully capture optimism arising from the complete model-development process. Biopsy sampling was heterogeneous, including 24-core sampling, and no MRI-targeted biopsies were performed. Restriction to exactly 12 systematic biopsy cores assessed the influence of biopsy extent but could not eliminate selection biases inherent in this surgical cohort. Selective lymph-node dissection precluded uniform inclusion of nodal status. Exclusion of 258 patients without MRI and 18 patients with inaccessible data may have introduced selection bias. Because variable-specific missingness information was unavailable for the latter group, complete-case status does not establish representativeness of the screened population. External transportability and clinical net benefit remain unestablished.

## 5. Conclusions

In this retrospective cohort of men diagnosed through systematic biopsy and treated with radical prostatectomy, adding PI-RADS findings from routine 1.5-T mpMRI to clinical and systematicbiopsy variables may provide only limited additional discrimination for adverse pathology. Although PI-RADS ≥ 4 remained independently associated with adverse pathology, the observed improvement over a model incorporating age, MRI-derived PSAD, biopsy Grade Group, and quantitative tumour burden was small and uncertain. These findings relate specifically to the incremental contribution of PI-RADS in the studied 1.5-T setting and should not be directly extrapolated to other imaging or biopsy pathways. External validation and assessment of clinical net benefit are warranted.

## Figures and Tables

**Table 1:** Baseline demographic, clinical, mpMRI, and systematicbiopsy characteristics stratified by adverse pathology at radical prostatectomy.

Variable	Overall (*n* = 284)	No Adverse Pathology (*n* = 141)	Adverse Pathology (*n* = 143)	*p* Value
**Demographic and clinical variables**				
Age, years	65 (60–68)	64 (59–67)	66 (62–70)	<0.001
PSA, ng/mL	7.8 (5.6–11.0)	7.2 (5.3–9.9)	9.0 (5.9–14.0)	0.002
PSAD, ng/mL/cm^3^	0.18 (0.12–0.26)	0.15 (0.10–0.22)	0.20 (0.13–0.32)	<0.001
Prostate volume, mL	46 (35–65)	50 (35–70)	43 (35–59)	0.024
Clinical T stage				0.184
cT1c	150 (52.8%)	82 (58.2%)	68 (47.6%)	
cT2a	97 (34.2%)	46 (32.6%)	51 (35.7%)	
cT2b	28 (9.9%)	10 (7.1%)	18 (12.6%)	
≥cT2c	9 (3.2%)	3 (2.1%)	6 (4.2%)	
**Magnetic resonance imaging variables**				
PI-RADS category				0.005
1	6 (2.1%)	3 (2.1%)	3 (2.1%)	
2	66 (23.2%)	38 (27.0%)	28 (19.6%)	
3	64 (22.5%)	40 (28.4%)	24 (16.8%)	
4	111 (39.1%)	50 (35.5%)	61 (42.7%)	
5	37 (13.0%)	10 (7.1%)	27 (18.9%)	
PI-RADS (≤3 vs. ≥4)				0.001
PI-RADS ≤ 3	136 (47.9%)	81 (57.4%)	55 (38.5%)	
PI-RADS ≥ 4	148 (52.1%)	60 (42.6%)	88 (61.5%)	
Number of MRI lesions	1 (0–1)	1 (0–1)	1 (1–1)	0.204
Largest MRI lesion, mm	10 (0–14)	9 (0–13)	11 (6–16)	0.007
MRI suspicion of EPE				0.007
Absent	270 (95.1%)	139 (98.6%)	131 (91.6%)	
Present	14 (4.9%)	2 (1.4%)	12 (8.4%)	
**Systematic biopsy variables**				
Total biopsy cores	12 (12–12)	12 (12–12)	12 (12–12)	0.842
Positive biopsy cores	4 (2–6)	3 (2–5)	5 (3–7)	<0.001
Positive cores, %	33.33 (16.67–50.00)	25.00 (16.67–41.67)	41.67 (25.00–58.33)	<0.001
Longest cancer length, mm	5 (3–8)	5 (3–7)	6 (4–9)	<0.001
Total core length, mm	108 (88–130)	109 (88–127)	108 (86–130)	0.803
Total cancer length, mm	13 (6–28.25)	10 (5–21)	20 (10–38)	<0.001
Tumour-to-core-length ratio, %	12.87 (5.54–26.19)	9.09 (4.76–16.92)	20.27 (10.34–34.36)	<0.001
Biopsy Grade Group				<0.001
1	135 (47.5%)	94 (66.7%)	41 (28.7%)	
2	86 (30.3%)	42 (29.8%)	44 (30.8%)	
3	27 (9.5%)	5 (3.5%)	22 (15.4%)	
4	28 (9.9%)	0 (0.0%)	28 (19.6%)	
5	8 (2.8%)	0 (0.0%)	8 (5.6%)	
Bilateral tumour on biopsy				0.089
Absent	165 (58.1%)	89 (63.1%)	76 (53.1%)	
Present	119 (41.9%)	52 (36.9%)	67 (46.9%)	
Perineural invasion				<0.001
Absent	241 (84.9%)	131 (92.9%)	110 (76.9%)	
Present	43 (15.1%)	10 (7.1%)	33 (23.1%)	
High-grade PIN				0.278
Absent	259 (91.2%)	126 (89.4%)	133 (93.0%)	
Present	25 (8.8%)	15 (10.6%)	10 (7.0%)	

Data are presented as median (Q1–Q3) for continuous variables and number (percentage) for categorical variables. Continuous variables were compared using the Mann–Whitney U test with tie and continuity corrections. Categorical variables were compared using Pearson’s chi-square test or Fisher’s exact test for sparse 2 × 2 tables, as appropriate. For sparse multi-category tables, a conditional Monte Carlo Pearson test (100,000 samples, fixed margins) was applied. For categorical variables with more than two levels, the reported *p* values represent overall between-group comparisons. Adverse pathology was defined as radical prostatectomy ISUP Grade Group ≥ 3 and/or pathological stage ≥ pT3a. PSAD was calculated as serum PSA divided by mpMRI-derived prostate volume. Abbreviations: EPE, extraprostatic extension; ISUP, International Society of Urological Pathology; mpMRI, multiparametric magnetic resonance imaging; PI-RADS, Prostate Imaging Reporting and Data System; PIN, prostatic intraepithelial neoplasia; PSA, prostate-specific antigen; PSAD, prostate-specific antigen density.

**Table 2:** Univariable logistic regression analysis of factors associated with adverse pathology at radical prostatectomy.

**Variable**	**OR**	**95% CI**	***p*** **Value**
**Clinical characteristics**			
Age, per 1-year increase	1.074	1.031–1.118	0.001
PSA, per 1 ng/mL increase	1.097	1.045–1.152	<0.001
PSAD, per 0.10 ng/mL/cm^3^ increase	1.478	1.223–1.786	<0.001
Prostate volume, per 1 mL increase	0.993	0.985–1.003	0.159
Clinical T-stageoverall effect (reference: T1c)			0.192
T2a vs. T1c	1.337	0.801–2.231	0.266
T2b vs. T1c	2.171	0.940–5.014	0.070
≥T2c vs. T1c	2.412	0.581–10.005	0.225
**Magnetic resonance imaging findings**			
PI-RADS ≥ 4, vs. PI-RADS ≤ 3	2.160	1.344–3.471	0.001
Number of MRI lesions, per additional lesion	1.209	0.900–1.624	0.208
Largest lesion diameter, per 1 mm increase	1.039	1.009–1.071	0.011
MRI suspicion of EPE, present vs. absent	6.366	1.398–28.988	0.017
**Histopathological findings at systematic biopsy**			
Total biopsy cores, per additional core	0.998	0.925–1.077	0.956
Positive biopsy cores, per additional core	1.234	1.126–1.352	<0.001
Positive biopsy core percentage, per 1% increase	1.026	1.015–1.038	<0.001
Longest cancer length, per 1 mm increase	1.101	1.029–1.178	0.005
Total cancer length, per 1 mm increase	1.029	1.015–1.043	<0.001
Tumour/core length ratio, per 1% increase	1.036	1.020–1.053	<0.001
Biopsy Grade Group 3–5, vs. Grade Group 1–2	18.560	7.157–48.129	<0.001
Bilateral disease on biopsy, present vs. absent	1.509	0.939–2.424	0.089
Perineural invasion, present vs. absent	3.930	1.854–8.333	<0.001
High-grade PIN, present vs. absent	0.632	0.274–1.458	0.282

Derived from unpenalized univariable logistic regression. ORs, 95% CIs, and two-sided *p* values were calculated using Wald tests. Clinical T-stage overall effect was evaluated using a 3-df Wald test. Increments for continuous predictors are specified in variable labels. Clinical T stage was analysed categorically with cT1c as the reference; the reported *p* value represents the overall effect of the variable. Adverse pathology was defined as radical prostatectomy ISUP Grade Group ≥ 3 and/or pathological stage ≥ pT3a. Abbreviations: CI, confidence interval; EPE, extraprostatic extension; ISUP, International Society of Urological Pathology; OR, odds ratio; PI-RADS, Prostate Imaging Reporting and Data System; PIN, prostatic intraepithelial neoplasia; PSA, prostate-specific antigen; PSAD, prostate-specific antigen density.

**Table 3:** Multivariable logistic regression models for adverse pathology at radical prostatectomy.

Variable	Model 1 Adjusted OR (95% CI)	*p* Value	Model 2 Adjusted OR (95% CI)	*p* Value	Model 3 Adjusted OR (95% CI)	*p* Value
Age, per 1-year increase	1.049 (1.002–1.099)	0.043	1.065 (1.020–1.111)	0.004	1.049 (1.001–1.100)	0.045
PSAD, per 0.10 ng/mL/cm^3^ increase	1.275 (1.044–1.558)	0.017	1.442 (1.192–1.745)	<0.001	1.274 (1.046–1.551)	0.016
PI-RADS ≥ 4, vs. PI-RADS ≤ 3	-	-	2.131 (1.289–3.523)	0.003	1.996 (1.150–3.465)	0.014
Biopsy Grade Group 3–5, vs. Grade Group 1–2	11.952 (4.491–31.808)	<0.001	-	-	11.830 (4.409–31.742)	<0.001
Tumour/core length ratio, per 1% increase	1.024 (1.007–1.042)	0.006	-	-	1.024 (1.006–1.042)	0.008
Apparent AUC (95% CI)	0.790 (0.737–0.843)	-	0.713 (0.653–0.773)	-	0.799 (0.747–0.851)	-

Values are presented as adjusted odds ratios with 95% confidence intervals. PSAD was scaled so that the reported ORs represent a 0.10 ng/mL/cm^3^ increase. Model 1 included age, PSAD, biopsy ISUP Grade Group, and tumour-to-core-length ratio. Model 2 included age, PSAD, and PI-RADS category. Model 3 incorporated all five predictors. All selected variables were entered simultaneously. Abbreviations: CI, confidence interval; ISUP, International Society of Urological Pathology; OR, odds ratio; PI-RADS, Prostate Imaging Reporting and Data System; PSAD, prostate-specific antigen density.

**Table 4:** Performance of the multivariable models for predicting adverse pathology.

**A. Discrimination and prediction error**					
**Model**	**Apparent AUC (95% CI)**		**Corrected AUC**	**Apparent Brier**	**Corrected Brier**
Model 1	0.790 (0.737–0.843)		0.781	0.184	0.191
Model 2	0.713 (0.653–0.773)		0.703	0.215	0.222
Model 3	0.799 (0.747–0.851)		0.787	0.178	0.187
**B. Calibration and usable bootstrap fits**					
**Model**	**Apparent Intercept**	**Apparent Slope**	**Corrected Intercept**	**Corrected Slope**	**Usable/Attempted**
Model 1	0.000	1.000	0.004	0.926	1987/2000
Model 2	0.000	1.000	0.002	0.947	2000/2000
Model 3	0.000	1.000	0.003	0.915	1987/2000

AUC CIs reflect DeLong intervals for apparent performance (not applicable to optimism-corrected AUCs). Calibration intercept denotes calibration-in-the-large (using prediction logit as offset); apparent intercept ≈ 0 and slope ≈ 1 derive from unpenalised model fitting on development data. Corrected estimates are adjusted by subtracting mean bootstrap optimism. Resampling was conducted using retained analysis records; models failing to yield a stable finite MLE were excluded and recorded. Lower Brier scores indicate superior performance. The primary M3 − M1 AUC difference is 0.009 (95% CI: −0.015 to 0.033); complete paired differences are presented in Table 5.

**Table 5:** Paired differences in apparent AUC.

Comparison	Apparent ΔAUC (95% CI)	DeLong *p*
M1 − M2	0.077 (0.024 to 0.130)	0.004
M3 − M2	0.086 (0.042 to 0.129)	<0.001
M3 − M1	0.009 (−0.015 to 0.033)	0.469

Paired comparisons of model predictions derived from the same development sample are purely descriptive. A non-significant DeLong test between nested fitted models does not imply equivalence or absence of incremental value. The contribution of the PI-RADS coefficient was evaluated separately via nested likelihood-ratio analysis.

## Data Availability

The data presented in this study are available from the corresponding author upon reasonable request. The data are not publicly available due to patient privacy and ethical restrictions.

## Supplementary Materials

The following supporting information can be downloaded at https://www.mdpi.com/article/10.3390/curroncol33090554/s1. Table S1, model performance and adjusted PI-RADS associations for separate pathological grade and stage outcomes; Table S2, complete logistic-regression coefficients for the three primary models; Table S3, ordinal PI-RADS sensitivity analysis in the full analytic cohort and the exactly 12-core systematicbiopsy subgroup; Table S4, assessment of nonlinearity and clinical T-stage sensitivity analyses; Table S5, adjusted associations and bootstrap optimism-corrected performance of the three primary models in the exactly 12-core systematicbiopsy subgroup.

## Abbreviations

The following abbreviations are used in this manuscript:

ADC	Apparent diffusion coefficient
AUC	Area under the receiver operating characteristic curve
CI	Confidence interval
EPE	Extraprostatic extension
IQR	Interquartile range
ISUP	International Society of Urological Pathology
MRI	Magnetic resonance imaging
mpMRI	Multiparametric magnetic resonance imaging
OR	Odds ratio
PI-RADS	Prostate Imaging Reporting and Data System
PIN	Prostatic intraepithelial neoplasia
PSA	Prostate-specific antigen
PSAD	Prostate-specific antigen density
Q1	First quartile
Q3	Third quartile
SD	Standard deviation
SPSS	Statistical Package for the Social Sciences
STROBE	Strengthening the Reporting of Observational Studies in Epidemiology
TNM	Tumour–Node–Metastasis
TRUS	Transrectal ultrasound

## References

[1] Siegel R.L., Kratzer T.B., Wagle N.S., Sung H., Jemal A. (2026). Cancer statistics, 2026. CA A Cancer J. Clin..

[2] Cornford P., Bergh R.C.v.D., Briers E., Broeck T.V.D., Brunckhorst O., Darraugh J., Eberli D., De Meerleer G., De Santis M., Farolfi A. (2024). EAU-EANM-ESTRO-ESUR-ISUP-SIOG Guidelines on Prostate Cancer—2024 Update. Part I: Screening, Diagnosis, and Local Treatment with Curative Intent. Eur. Urol..

[3] van Leenders G.J., van der Kwast T.H., Grignon D.J., Evans A.J., Kristiansen G., Kweldam C.F., Litjens G., McKenney J.K., Melamed J., Mottet N. (2020). The 2019 International Society of Urological Pathology (ISUP) Consensus Conference on Grading of Prostatic Carcinoma. Am. J. Surg. Pathol..

[4] Turkbey B., Rosenkrantz A.B., Haider M.A., Padhani A.R., Villeirs G., Macura K.J., Tempany C.M., Choyke P.L., Cornud F., Margolis D.J. (2019). Prostate Imaging Reporting and Data System Version 2.1: 2019 Update of Prostate Imaging Reporting and Data System Version 2. Eur. Urol..

[5] Ahmed H.U., El-Shater Bosaily A., Brown L.C., Gabe R., Kaplan R., Parmar M.K., Collaco-Moraes Y., Ward K., Hindley R.G., Freeman A. (2017). Diagnostic accuracy of multi-parametric MRI and TRUS biopsy in prostate cancer (PROMIS): A paired validating confirmatory study. Lancet.

[6] Kasivisvanathan V., Rannikko A.S., Borghi M., Panebianco V., Mynderse L.A., Vaarala M.H., Briganti A., Budäus L., Hellawell G., Hindley R.G. (2018). ECISION Study Group Collaborators. MRI-Targeted or Standard Biopsy for Prostate-Cancer Diagnosis. N. Engl. J. Med..

[7] Rouvière O., Puech P., Renard-Penna R., Claudon M., Roy C., Mège-Lechevallier F., Decaussin-Petrucci M., Dubreuil-Chambardel M., Magaud L., Remontet L. (2019). Use of prostate systematic and targeted biopsy on the basis of multiparametric MRI in biopsy-naive patients (MRI-FIRST): A prospective, multicentre, paired diagnostic study. Lancet Oncol..

[8] Drost F.-J.H., Osses D.F., Nieboer D., Steyerberg E.W., Bangma C.H., Roobol M.J., Schoots I.G. (2019). Prostate MRI, with or without MRI-targeted biopsy, and systematic biopsy for detecting prostate cancer. Cochrane Database Syst. Rev..

[9] de Rooij M., Hamoen E.H., Witjes J.A., Barentsz J.O., Rovers M.M. (2016). Accuracy of Magnetic Resonance Imaging for Local Staging of Prostate Cancer: A Diagnostic Meta-analysis. Eur. Urol..

[10] Humke C., Hoeh B., Preisser F., Wenzel M., Welte M.N., Theissen L., Bodelle B., Koellermann J., Steuber T., Haese A. (2022). Concordance between Preoperative mpMRI and Pathological Stage and Its Influence on Nerve-Sparing Surgery in Patients with High-Risk Prostate Cancer. Curr. Oncol..

[11] Wibmer A.G., Kattan M.W., Alessandrino F., Baur A.D.J., Boesen L., Franco F.B., Bonekamp D., Campa R., Cash H., Catalá V. (2021). International Multi-Site Initiative to Develop an MRI-Inclusive Nomogram for Side-Specific Prediction of Extraprostatic Extension of Prostate Cancer. Cancers.

[12] Distler F.A., Radtke J.P., Bonekamp D., Kesch C., Schlemmer H.-P., Wieczorek K., Kirchner M., Pahernik S., Hohenfellner M., Hadaschik B.A. (2017). The Value of PSA Density in Combination with PI-RADS™ for the Accuracy of Prostate Cancer Prediction. J. Urol..

[13] Schoots I.G., Padhani A.R. (2020). Risk-adapted biopsy decision based on prostate magnetic resonance imaging and prostate-specific antigen density for enhanced biopsy avoidance in first prostate cancer diagnostic evaluation. BJU Int..

[14] de Rooij M., Israël B., Tummers M., Ahmed H.U., Barrett T., Giganti F., Hamm B., Løgager V., Padhani A., Panebianco V. (2020). ESUR/ESUI consensus statements on multi-parametric MRI for the detection of clinically significant prostate cancer: Quality requirements for image acquisition, interpretation and radiologists’ training. Eur. Radiol..

[15] Barrett T., de Rooij M., Giganti F., Allen C., Barentsz J.O., Padhani A.R. (2022). Quality checkpoints in the MRI-directed prostate cancer diagnostic pathway. Nat. Rev. Urol..

[16] Cheng Y., Zhang L., Wu X., Zou Y., Niu Y., Wang L. (2024). Impact of prostate MRI image quality on diagnostic performance for clinically significant prostate cancer (csPCa). Abdom. Radiol..

[17] Giganti F., Ng A., Asif A., Chan V.W.-S., Rossiter M., Nathan A., Khetrapal P., Dickinson L., Punwani S., Brew-Graves C. (2023). Global Variation in Magnetic Resonance Imaging Quality of the Prostate. Radiology.

[18] Malewski W., Milecki T., Tayara O., Poletajew S., Kryst P., Tokarczyk A., Nyk Ł. (2024). Role of Systematic Biopsy in the Era of Targeted Biopsy: A Review. Curr. Oncol..

[19] Collins G.S., Moons K.G.M., Dhiman P., Riley R.D., Beam A.L., Van Calster B., Ghassemi M., Liu X., Reitsma J.B., van Smeden M. (2024). TRIPOD + AI statement: Updated guidance for reporting clinical prediction models that use regression or machine learning methods. BMJ.

[20] Thompson J., van Leeuwen P., Moses D., Shnier R., Brenner P., Delprado W., Pulbrook M., Böhm M., Haynes A., Hayen A. (2016). The Diagnostic Performance of Multiparametric Magnetic Resonance Imaging to Detect Significant Prostate Cancer. J. Urol..

[21] Diamand R., Roche J.-B., Lievore E., Lacetera V., Chiacchio G., Beatrici V., Mastroianni R., Simone G., Windisch O., Benamran D. (2022). External Validation of Models for Prediction of Side-specific Extracapsular Extension in Prostate Cancer Patients Undergoing Radical Prostatectomy. Eur. Urol. Focus.

[22] Alves J.R., Muglia V.F., Lucchesi F.R., Faria R.A.O.G., Alcantara-Quispe C., Vazquez V.L., Reis R.B., Faria E.F. (2020). Independent external validation of nomogram to predict extracapsular extension in patients with prostate cancer. Eur. Radiol..

[23] Soeterik T.F., van Melick H.H., Dijksman L.M., Biesma D.H., Witjes J.A., van Basten J.-P.A. (2021). Multiparametric Magnetic Resonance Imaging Should Be Preferred Over Digital Rectal Examination for Prostate Cancer Local Staging and Disease Risk Classification. Urology.

[24] Faiena I., Salmasi A., Mendhiratta N., Markovic D., Ahuja P., Hsu W., Elashoff D.A., Raman S.S., Reiter R.E. (2019). PI-RADS Version 2 Category on 3 Tesla Multiparametric Prostate Magnetic Resonance Imaging Predicts Oncologic Outcomes in Gleason 3 + 4 Prostate Cancer on Biopsy. J. Urol..

[25] Vargas H.A., Hotker A.M., Goldman D.A., Moskowitz C.S., Gondo T., Matsumoto K., Ehdaie B., Woo S., Fine S.W., Reuter V.E. (2016). Updated prostate imaging reporting and data system (PIRADS v2) recommendations for the detection of clinically significant prostate cancer using multiparametric MRI: Critical evaluation using whole-mount pathology as standard of reference. Eur. Radiol..

