# Supplementary material for: Incremental Value of PI-RADS v2.1 for Adverse Pathology at Radical Prostatectomy: A Retrospective 1.5-T MRI Cohort
Source: Curr Oncol. 2026 Sep 11;33(9):554. doi: 10.3390/curroncol33090554 (PMC13605579; doi:10.3390/curroncol33090554)
Supplement: Supplementary file 1 [file curroncol-33-00554-s001.zip › curroncol-4512080-supplementary.pdf]

Table S1. Model performance and adjusted PI-RADS associations for separate pathological grade and stage outcomes in the full analytic cohort.

Outcome / model	Events, n	Apparent AUC (95% CI)	Optimism-corrected AUC
Grade Group ≥3 / M1	83	0.839 (0.781–0.897)	0.826
Grade Group ≥3 / M2	83	0.728 (0.661–0.796)	0.717
Grade Group ≥3 / M3	83	0.852 (0.798–0.906)	0.836
Stage ≥pT3a / M1	123	0.785 (0.729–0.840)	0.775
Stage ≥pT3a / M2	123	0.738 (0.679–0.797)	0.728
Stage ≥pT3a / M3	123	0.788 (0.734–0.843)	0.775

For prostatectomy Grade Group ≥3, the M3−M1 apparent ΔAUC was 0.013 (95% CI, −0.001 to 0.027; descriptive paired DeLong p = 0.065), and the adjusted OR for PI-RADS ≥4 versus ≤3 was 1.462 (95% CI, 0.743–2.877; Wald p = 0.271). For pathological stage ≥pT3a, the corresponding ΔAUC was 0.003 (95% CI, −0.012 to 0.019; descriptive paired DeLong p = 0.662), and the adjusted PI-RADS OR was 1.659 (95% CI, 0.958–2.872; Wald p = 0.071).

The full analytic cohort included 284 patients. The grade outcome was prostatectomy Grade Group ≥3, and the stage outcome was pathological stage ≥pT3a. "Events" denotes the number of patients meeting the corresponding outcome definition. Model 1 included age, MRI-derived PSA density (PSAD), biopsy Grade Group 3–5 versus 1–2, and the tumour-to-core-length ratio. Model 2 included age, MRI-derived PSAD, and PI-RADS ≥4 versus ≤3. Model 3 included the predictors in Model 1 plus PI-RADS ≥4 versus ≤3. Models were refitted separately for each outcome. AUC values are apparent AUCs with 95% confidence intervals calculated using DeLong's method; corrected AUCs are optimism-corrected estimates obtained by bootstrap internal validation. Adjusted PI-RADS odds ratios and Wald p values are reported from Model 3 and compare PI-RADS ≥4 with ≤3. Paired DeLong comparisons are descriptive because the models were developed and evaluated in the same cohort. Both outcome components may occur in the same patient. Differences between outcome-specific p values do not demonstrate different PI-RADS associations with grade and stage.

Table S2. Complete logistic-regression coefficients for the three primary models in the full analytic cohort.

Model	Term	Beta	SE	Wald p
M1	Intercept	-4.409470	1.542027	0.004
	Age, per year	0.047885	0.023654	0.043
	PSAD, per 0.10 ng/mL/cm³	0.243113	0.102075	0.017
	Biopsy Grade Group 3–5 vs 1–2	2.480928	0.499396	<0.001
	Tumour-to-core-length ratio, per percentage point	0.024018	0.008779	0.006
M2	Intercept	-5.192610	1.416941	<0.001
	Age, per year	0.062678	0.021667	0.004
	PSAD, per 0.10 ng/mL/cm³	0.366238	0.097104	<0.001
	PI-RADS ≥4 vs ≤3	0.756819	0.256439	0.003
M3	Intercept	-4.765185	1.571047	0.002
	Age, per year	0.048029	0.023922	0.045
	PSAD, per 0.10 ng/mL/cm³	0.241842	0.100491	0.016
	Biopsy Grade Group 3–5 vs 1–2	2.470605	0.503605	<0.001
	Tumour-to-core-length ratio, per percentage point	0.023644	0.008878	0.008
	PI-RADS ≥4 vs ≤3	0.691314	0.281300	0.014

β denotes the logistic-regression coefficient on the log-odds scale; SE denotes the standard error; and p denotes the two-sided Wald p value. Predicted probability = 1/[1 + exp(-η)], w**here η = intercept + Σ(βj × Xj).** Age was entered in years. PSAD was scaled as PSAD/(0.10 ng/mL/cm³), and the tumour-to-core-length ratio was entered in percentage points. Binary indicators were coded as 0 for the reference category and 1 for the comparison category: biopsy Grade Group 3–5 versus 1–2 and PI-*RADS ≥4 versus ≤3. Model 1 included age, MRI-derived PSAD, biopsy Grade Group, and tumour-to-core-length ratio; Model 2 included age, MRI-derived PSAD, and PI-RADS; Model 3 included the predictors of Model 1 plus PI-RADS. The coefficients shown are from the fitted full-cohort models and are not bootstrap-corrected coefficients.*

Table S3. Ordinal PI-RADS sensitivity analysis in the full analytic cohort and the exactly 12-core systematic-biopsy subgroup.

Measure	Full cohort (n = 284)	Exactly 12 cores (n = 255)
**A. Adverse pathology by PI-RADS category, n/N (%)**		
1	3/6 (50.0)	2/4 (50.0)
2	28/66 (42.4)	24/59 (40.7)
3	24/64 (37.5)	23/58 (39.7)
4	61/111 (55.0)	57/100 (57.0)
5	27/37 (73.0)	24/34 (70.6)
**B. Combined model with ordinal PI-RADS**		
PI-RADS adjusted OR (95% CI)	1.395 (1.066–1.827)	1.454 (1.088–1.942)
Wald p for PI-RADS	0.015	0.011
Apparent AUC (95% CI)	0.799 (0.747–0.852)	0.808 (0.754–0.861)
Optimism-corrected AUC	0.786	0.795
Apparent ΔAUC vs. Model 1 (95% CI)	0.009 (−0.014 to 0.032)	0.012 (−0.014 to 0.037)
Descriptive DeLong p for ΔAUC	0.452	0.368
Likelihood-ratio χ^2 (1 df); p	6.054; p = 0.014	6.636; p = 0.010
Corrected calibration intercept	0.004	0.003
Corrected calibration slope	0.913	0.921
Corrected Brier score	0.187	0.184
Usable / attempted bootstrap fits	1987/2000	1985/2000

Adverse pathology was defined as prostatectomy Grade Group ≥3 and/or pathological stage ≥pT3a. Panel A reports the number of patients with adverse pathology among the total number of patients in each PI-RADS category, with percentages calculated within category.
Model 1 included age, MRI-derived PSAD, biopsy Grade Group 3–5 versus 1–2, and the tumour-to-core-length ratio. The ordinal combined model consisted of Model 1 plus PI-RADS coded as a single numeric predictor from 1 to 5; the binary PI-RADS term was not included simultaneously. The reported odds ratio represents a one-category increase in PI-RADS and assumes a constant change in log odds across categories. Models were refitted separately in the full cohort and the exactly 12-core subgroup.
AUC and ΔAUC values are apparent estimates. Their 95% confidence intervals were calculated using DeLong's method; ΔAUC intervals were based on paired predictions. The Wald p value evaluates the ordinal PI-RADS coefficient, whereas the likelihood-ratio test evaluates the addition of the ordinal PI-RADS term to Model 1. Because the models were fitted and evaluated in the same cohort, paired DeLong comparisons are descriptive.
Optimism-corrected AUC, calibration, and Brier-score estimates were obtained using 2,000 nonparametric bootstrap resamples with model refitting and fixed predictor sets. Bootstrap fits without stable finite estimates were excluded from the corresponding corrected estimates and counted in the reported usable-fits ratio. The calibration intercept denotes calibration-in-the-large. Lower Brier scores indicate lower prediction error.

Table S4. Assessment of nonlinearity and clinical T-stage sensitivity analyses in the full analytic cohort.

Likelihood-ratio comparison	χ^2	df	p
All nonlinear terms jointly	2.578	3	0.461
Age nonlinear term	0.421	1	0.516
PSAD nonlinear term	0.101	1	0.751
Tumour-to-core-length ratio nonlinear term	1.940	1	0.164

Restricted cubic-spline analyses were fitted within Model 3 using knots at the 10th, 50th, and 90th percentiles of the full analytic cohort. Each continuous predictor contributed one linear and one nonlinear basis term. The joint likelihood-ratio comparison tested the spline version of Model 3 against the corresponding model with linear terms only. Individual nonlinear terms were tested conditionally on the remaining spline terms. Knot locations were fixed at the cohort-specific values during bootstrap resampling.

Predictor	10th percentile	50th percentile	90th percentile
Age (years)	56.000000	65.000000	72.000000
PSAD/(0.10 ng/mL/cm^3)	0.734733	1.763305	4.299000
Tumour-to-core-length ratio (percentage points)	3.188787	12.870945	43.396166

The additional clinical T-stage analysis added cT≥2 versus cT1c to Model 3. The likelihood-ratio comparison between the cT-adjusted model and Model 3 yielded $\chi^2(1)=0.461$, $p=0.497$. In the recorded source variable, the category ≥cT2c did not distinguish cT2c from higher clinical stages, including cT3a; therefore, the sensitivity analysis used the broader cT≥2 versus cT1c contrast. This analysis was an additional clinical-predictor sensitivity analysis and was not a validation of an external risk score.

PI-RADS increment after additional clinical T-stage adjustment

Comparison	Apparent ΔAUC (95% CI)	Descriptive paired DeLong p
cT-adjusted Model 3 − cT-adjusted Model 1	0.009 (-0.014 to 0.033)	0.441

The cT-adjusted Models 1 and 3 denote the corresponding primary models with cT≥2 versus cT1c added. Paired DeLong comparisons are descriptive because the models were fitted and evaluated in the same development cohort; a nonsignificant result does not establish equivalence or absence of added information. The likelihood-ratio test described above evaluates the addition of clinical T stage to Model 3 and is distinct from the DeLong comparison of the PI-RADS increment.

Table S5. Adjusted associations and bootstrap optimism-corrected performance of the three primary models in the exactly 12-core systematic-biopsy subgroup.

Measure	Model 1	Model 2	Model 3
**A. Adjusted OR (95% CI); Wald p**			
Age, per year	1.054<br>(1.004–1.106)<br>p = 0.035	1.070<br>(1.024–1.118)<br>p = 0.003	1.054<br>(1.004–1.107)<br>p = 0.035
PSAD, per 0.10 ng/mL/cm³	1.210<br>(0.997–1.467)<br>p = 0.054	1.385<br>(1.141–1.682)<br>p = 0.001	1.212<br>(1.002–1.466)<br>p = 0.047
PI-RADS ≥4 vs ≤3	—	2.228<br>(1.312–3.781)<br>p = 0.003	2.159<br>(1.199–3.887)<br>p = 0.010
Biopsy Grade Group 3–5 vs 1–2	11.896<br>(4.443–31.850)<br>p < 0.001	—	11.858<br>(4.384–32.077)<br>p < 0.001
Tumour-to-core-length ratio, per percentage point	1.026<br>(1.008–1.045)<br>p = 0.004	—	1.027<br>(1.008–1.045)<br>p = 0.004
**B. Discrimination, calibration and prediction error**			
Apparent AUC (95% CI)	0.796<br>(0.741–0.851)	0.712<br>(0.649–0.776)	0.807<br>(0.753–0.861)
Optimism-corrected AUC	0.787	0.702	0.795
Brier score: apparent / corrected	0.182 / 0.189	0.216 / 0.223	0.175 / 0.184
Corrected calibration intercept	0.002	0.001	0.002
Corrected calibration slope	0.932	0.944	0.921
Usable / attempted bootstrap fits	1985/2000	2000/2000	1985/2000
**C. Paired comparisons of apparent discrimination**			
**Comparison**	**Model 1 − Model 2**	**Model 3 − Model 2**	**Model 3 − Model 1**
ΔAUC (95% CI)	0.083<br>(0.025 to 0.142)	0.095<br>(0.046 to 0.143)	0.011<br>(−0.015 to 0.038)
Descriptive DeLong p	0.005	<0.001	0.413

The subgroup included 255 patients, of whom 130 had adverse pathology defined as prostatectomy Grade Group ≥3 and/or pathological stage ≥pT3a. The analysis was restricted to patients who underwent exactly 12 systematic biopsy cores, and all models were refitted within this subgroup.

Model 1 included age, MRI-derived PSAD, biopsy Grade Group 3–5 versus 1–2, and the tumour-to-core-length ratio. Model 2 included age, MRI-derived PSAD, and PI-RADS ≥4 versus ≤3. Model 3 included the predictors of Model 1 plus PI-RADS ≥4 versus ≤3. —, predictor not included; OR, odds ratio; CI, confidence interval; AUC, area under the receiver operating characteristic curve.

OR confidence intervals and p values are based on two-sided Wald estimates. Apparent AUCs and ΔAUCs are reported with 95% confidence intervals calculated using DeLong's method; ΔAUC intervals are based on paired predictions. Paired comparisons are descriptive, particularly for nested models fitted and evaluated in the same cohort. Differences were calculated from unrounded estimates.

Bootstrap internal validation used 2,000 nonparametric resamples with replacement from the 12-core subgroup, with refitting of each model and fixed predictor sets. The number of usable bootstrap fits is reported as usable/attempted fits. Optimism-corrected AUCs, calibration measures, and Brier scores were obtained from the bootstrap procedure. Calibration intercept denotes calibration-in-the-large, and lower Brier scores indicate lower prediction error.
